# Can Infants Perceive and Learn New Information from Extended Reality?

**DOI:** 10.1111/desc.70111

**Published:** 2026-01-06

**Authors:** Liquan Liu, Brayden Condie, Jasmine Sutton, Sharmin Saba, Ashleigh Blackwell, Faiza Humaira, Rakhshinda Shoaib, David Arness, Tomas Trescak

**Affiliations:** ^1^ Graduate School of Health University of Technology Sydney Sydney Australia; ^2^ School of Psychology Western Sydney University Sydney Australia; ^3^ MARCS Institute for Brain and Behaviour Western Sydney University Sydney Australia; ^4^ Center for Multilingualism in Society Across the Lifespan University of Oslo Oslo Norway; ^5^ Centre of Excellence for the Dynamics of Language Australian Research Council Canberra Australia; ^6^ School of Computer, Data and Mathematical Sciences Western Sydney University Sydney Australia

## Abstract

As global societies increasingly embrace digital technologies, their integration into early childhood education becomes crucial for achieving United Nations sustainable developmental goals. The present study investigates whether extended reality (XR) environments effectively support infants' perception and learning capabilities. A total of 144 Australian infants aged 6–12 and 18–24 months were tested on their abilities to discriminate and learn non‐native Mandarin tones under one of the three conditions, Live (face‐to‐face interaction), XR‐Live (real‐time virtual interaction), and XR‐Recorded (pre‐recorded virtual interaction). Infants successfully discriminated the tone contrast, with perception outcomes influenced by age and condition: older infants exhibited robust discrimination in both XR conditions, and long looking time in the Live condition. In object‐label association, infants learned equally well in Live and both XR conditions, influenced by age and tone complexity—older infants learned better overall, particularly the acoustically complex dipping tone. Findings highlight XR's potential to effectively support sustainable cognitive and linguistic development, underscoring its promise for future integration in early educational and developmental interventions.

**Summary:**

- Second‐year infants demonstrated enhanced perceptual discrimination of non‐native speech after targeted exposure in XR environments.
- Second‐year infants showed robust attention to non‐native speech following face‐to‐face interaction with a native speaker of that language.
- Second‐year infants demonstrated equivalent learning of non‐native speech across XR and face‐to‐face conditions, highlighting XR's potential to support early language development.
- Infants showed stronger learning of the acoustically complex dipping tone than the rising tone, with older infants outperforming their younger counterparts.

## 1 Introduction

Modern technology has become an integral part of daily life, reshaping child‐rearing practices and influencing how infants experience the world. Despite the growing presence of digital media, our understanding of infant‐technology interaction remains limited. Infants, lacking independent mobility, rely on caregivers not only for basic needs but also for access to environments essential for learning. Situations such as COVID‐19 can restrict these opportunities, prompting caregivers to turn to screen media—despite evidence suggesting its limited interactivity and reduced effectiveness in supporting learning in early childhood (e.g., DeLoache et al. 2010; Kuhl et al. 2003). This study explores whether extended reality (XR, Rauschnabel et al. 2022), the rapidly emerging yet under‐researched new reality format, can support infants’ ability to perceive and abstract new information.

Science in the past decade has ubiquitously pointed to the importance of early life experience on human learning and development. The first 1000 days mark the golden age of learning and foundation of children's cognitive abilities and future success (Romeo et al. 2018). Perceptual categories, neural paths, and strategies for learning world knowledge are shaped by infants’ experience before and since birth (Werker and Hensch 2015). Adoptees retain implicit memory of their birth language, even when adopted in the first year of life (Pierce et al. 2014).

Theoretical frameworks, including perceptual narrowing (Kuhl et al. 2008; Lewkowicz and Ghazanfar 2009; Scott et al. 2007) critical period (Werker and Hensch 2015), and flexibility‐based (Bahrick et al. 2004; Werker and Curtin 2005) hypotheses, mark the emergence of multisensory systems and the developmental trajectory of language. Taking lexical tone as an example, tone is defined as linguistic pitch used to distinguish word meaning. Different from intonation which conveys pragmatic or emotional functions on a phrasal level, tone is contrastive at the lexical level (Yip 2002). In the course of perceptual development, newborn and young infants initially possess a broad ability to distinguish a wide range of phonetic differences such as word‐level pitch contrasts (Nazzi et al. 1998). Over the first year of life, atonal language‐learning infants become increasingly attuned to the specific phonemes of their native language, resulting in reduced sensitivity to non‐native tone contrasts (Cabrera et al. 2015; Fikkert et al. 2020; Mattock and Burnham 2006).

In the developmental trajectory of learning words, the sounds considered as permissible labels appear flexible early on and are gradually restricted to phonemes of infants’ native languages in the second year of life (Woodward and Hoyne 1999). Infants as young as 4 months linked novel auditory labels to visual referents after a single exposure, and later recognised these associations (Saksida and Langus 2024). Nine‐month‐old English‐learning infants demonstrated ability to associate non‐native tones with objects (Yeung and Werker 2009; Yeung et al. 2014). Similarly, Dutch‐learning and English‐learning infants associated novel objects with words contrasted only in tone at 14 months but can no longer do so by 17–19 months (Burnham et al. 2018; Hay et al. 2019, 2015; Liu and Kager 2018). In other words, by the end of second year, atonal language‐learning infants no longer treat tone as lexically contrastive, reflecting a developmental shift toward native phonological categories.

Nevertheless, findings on non‐native tone perceptual development are mixed, with evidence showing other patterns from sensitivity maintenance (Chen et al. 2022; Kalashnikova et al. 2024), to increase (Chen et al. 2017) and recovery (Götz et al. 2018; Liu and Kager 2014) as infants grow. The developmental trajectory is thus argued to be modulated by factors such as perceptual salience (Burnham and Singh 2018) and language experience (Reid et al. 2015), among others. In terms of perceptual salience, not all tones/tone contrasts are perceived in the same way. Some are more easily discriminated (Huang and Johnson 2011; Liu et al. 2022) or learned (Li and Thompson 1977) than others regardless of listeners’ age and language background. The development of infants’ tone processing has been shown as a function of tone contrasts. For example, Mandarin features four primary tones: level, rising, dipping, and falling. The level‐falling tone contrast has been shown to be easier to process than the rising‐dipping contrast by the end of the second year (Cheng and Lee 2018).

Moreover, listeners’ native language experience can also modulate perception and learning outcomes. For instance, English listeners’ perception of the rising tone in Mandarin has been argued to be interfered with by their existing knowledge of interrogative intonation, as both pitch contours share similar rising patterns (So and Best 2011). In addition, 14‐month‐old English‐learning infants showed successful associative word learning of Mandarin tone contrasts but only when one of the labels is rising tone (Hay et al. 2019). The authors suggest that exposure to and production of native language prosody may cause infants to over‐interpret the rising pitch in distinguishing words. These cross‐linguistic interference effects are consistent with theoretical frameworks (e.g., Best 1994).

Another critical aspect influencing early childhood learning and development lies in environmental input, and modern technology such as screen media‐induced learning in early childhood has received much attention and heated discussion. With the widespread availability of technology, screen media has become a significant source of language exposure for young children (Hartshorne et al. 2021; Werker 2024). Screen media includes a range of technologies characterized by mediated, rather than direct interaction with a person or event (Jing et al. 2023). As many young children exceed recommended limits in screen time (Madigan et al. 2020; Vandewater et al. 2007), the high rate of screen media consumption has made its impact on language development a topic of considerable debate and research.

Empirical evidence suggests that digital media does not facilitate learning as well as face‐to‐face interactions. When comparing different contexts for language learning, face‐to‐face interactions appear more successful or efficient in teaching infants new words and supporting overall language development than screen‐based technology (Adelantado‐Renau et al. 2019; Brushe et al. 2024; DeLoache et al. 2010; Robb et al. 2009). In Kuhl et al. (2003), 9‐month‐old English‐learning infants were randomly assigned to one of three environments: live interaction with native Mandarin speakers, exposure to pre‐recorded video, or audio‐only recordings, with identical linguistic content presented across all conditions. Only infants in the live interaction group showed improved Mandarin speech perception. Findings are consistent with a meta‐analysis studying 122 independent effect sizes across 59 reports involving children aged 0–6 years, resulting in a video deficit effect showing young children learn significantly less from screen media than from direct, face‐to‐face interactions (Strouse and Samson 2021). A negative association has been reported between children's screen media exposure and their vocabulary development (Sundqvist et al. 2024), and infants' imitation of media‐based events appears to be limited by developmental changes in memory flexibility (Barr 2013). Although it has been argued that learning does not occur through televised/pre‐recorded stimulus (Hakuno et al. 2017), it is unclear whether the essential factor lies in the physical presence of an adult or social contingency—the reciprocal exchange between an adult and an infant. In a later study, toddlers aged 24–30 months were randomly assigned to one of three environments: live interaction with an adult, socially contingent video chat (e.g., Skype), and noncontingent pre‐recorded video (Roseberry et al. 2014). A preferential looking paradigm was used to measure visual fixation to two images presented side‐by‐side while exposed to an auditory stimulus previously paired with one of the two visual stimuli. Only children in the socially contingent conditions with real‐time, reciprocal communication demonstrated successful learning. Thus, applications like Skype facilitate a social component similar to live interactions (Krcmar 2011). Other studies have attributed negative learning outcomes to reduced engagment (e.g., Strouse et al. 2018) or impaired language perception (e.g., Linebarger and Vaala 2010). Findings highlight the importance of social interaction since birth (Endevelt‐Shapira et al. 2024), adhering to social cognition hypotheses (e.g., Tomasello 2003) and sociocultural theories (e.g., Vygotsky 1962). Enriched with cues such as eye contact, facial expressions and gestures, social interaction enhances infants’ learning experience by providing them with a multi‐sensory context for language input (Kuhl 2007; Tsui et al. 2019). Having said that, controlling for perceptual differences such as the size of the visible partner and the visual angle, another study found face‐to‐face interaction results in greater word learning than live screen‐mediated interaction among children aged 24‐36 months (Myers et al. 2019). This raises the question of whether improving visual clarity or immersive experience may enhance learning via screen‐based technology.

Meanwhile, research on children's learning from screen media has yielded mixed results, as some studies indicate that young children can acquire vocabulary through screens (Scofield and Williams 2009). For example, a recent meta‐analysis has reported a small but positive benefit of screen exposure on vocabulary development (Jing et al. 2023). These findings suggest that the effectiveness of digital media may depend less on its mere presence and more on how it is used, the content provided, and relevant contextual or individual factors.

Although traditional screens continue to play a vital role in education and communication, growing attention is being directed toward XR technologies for their capacity to simulate real‐world interactions (Wang et al. 2024). XR, a general term for new reality formats that involve virtual elements generated by computer technology, offers ingenious and viable learning possibilities (Krueger 1991). From augmented, extended, virtual to mixed reality, XR aims to provide immersive, context‐rich environments that resemble real‐life experiences (Milgram and Kishino 1994; Rauschnabel et al. 2022). Understanding how XR can influence early language learning could lead to the development of innovative educational tools that leverage immersive technology to support optimal development in children (Chen et al. 2022). While no prior study has explored the infant‐XR relationship, research involving older age groups and various educational contexts has demonstrated facilitation effects in XR environments. For instance, compared to traditional classroom learning of a second language, virtual reality‐based learning has yielded increased engagement, a 20% increase in vocabulary skills, and improvement in speaking and pronunciation of 6–8‐year‐old children (Si 2015).

XR features in vividness and interactivity (Steuer 1992). Vividness, or representational richness, refers to the way in which a mediated environment presents information to human senses. It is dependent on the technical characteristics of the presentation medium, and modern technologies push the sensory breadth and depth of mediated experience to the representational goal that is indistinguishable from the real‐world counterparts when the difference between reality and XR representation is blurred. To investigate this feature, this study examined whether the mode of presentation—face‐to‐face versus live projection—would yield comparable outcomes in infant perception and learning.

Interactivity—the extent to which users can engage with and alter the form and content of a mediated environment in real time—is another key feature of human‐XR interaction. This issue becomes particularly salient in traditional screen media, where limited interactivity may underlie the video deficit effect as well as inefficient learning transfer between objects presented by screen and real objects in the physical world (Zack and Barr 2016). Access to social information, such as parental responsiveness (Tamis‐LeMonda et al. 2014), real‐time contingent feedback, and referential social cues like eye contact and gaze following (Strouse et al. 2018), is often absent in pre‐recorded contexts. To address this, the present study also examined whether pre‐recorded videos and live interactions delivered via XR produce comparable learning outcomes.

As arguably the most critical stage of development, infancy presents an unexplored frontier for understanding how XR technologies interact with and impact early learning. In light of the growing inevitability of digital media exposure and the absence of prior research on XR‐mediated interactions in early childhood, it is both timely and essential to examine how immersive technologies might facilitate infant learning through novel modes of information delivery. The present study was the first to investigate whether infants can perceive and learn new information via XR. Detailed research questions include: Specifically, do infants perceive and acquire new information from XR to the same extent as they do in real life? What is the developmental trajectory of learning from XR, and how do learning targets play a role in this process?

To answer these questions, first‐ and second‐year Australian infants were exposed to two legal non‐words contrasted in Mandarin tones embedded in a play session in Live, XR‐Live, or XR‐Recorded conditions. Infants were subsequently tested on their object‐label association ability and perception of tonal differences. Two age groups were selected spanning the key period of perceptual attunement: A younger cohort (6–12 months) within the sensitive period for non‐native phonetic discrimination, and an older cohort (18–24 months) targeting the decline of associative word learning of non‐native contrasts (Werker and Hensch 2015).

Due to the difficulties in fitting head‐mounted headsets (HMD) for 3D projection on young children, its weight, along with ethical concerns such as the ability to easily withdraw (e.g., removing an HMD), research with infants and toddlers must consider alternative mediums. Visual projections can provide immersive experience by filling the infant's visual field, effectively replicating the qualities of HMD without requiring cumbersome or uncomfortable wearable devices. As infants’ visual capacity is still developing, a 2D projection can effectively simulate an immersive environment (Cárdenas‐Robledo et al. 2022). Additionally, XR‐based educational approaches hold potential environmental advantages, significantly reducing the need for physical resources and minimizing travel‐related emissions associated with conventional face‐to‐face educational interactions. As the global community seeks environmentally sustainable solutions in education, XR presents itself as a compelling alternative aligned with global sustainability frameworks.

We predicted that infants could perceive and acquire new information from XR, though they may not learn as efficiently as in real life (DeLoache et al. 2010). Given prior mixed findings in perception and learning of non‐native tones along the developmental trajectory (Fikkert et al. 2020), the effect of age was kept open for exploration. As for the learning targets, a number of factors may be in play. While perceiving and learning acoustically more complex tones (e.g., dipping tone in Mandarin) may be naturally more challenging, it may be perceptually more salient, whereas tones with similar contours as pitch functions in infants’ native language inventory (e.g., rising tone in Mandarin and interrogative intonation in Australian English) may lead to an interference effect which affects perception and lexical coding.

## 2 Methods

### 2.1 Participants

The final sample consisted of 144 Australian English‐learning infants from middle‐income, predominantly Caucasian families in the suburban Western Sydney region, split evenly between 1‐year‐olds (6–12 months, *N* = 72, *M* = 8.38, SD = 2.11, Female = 35) and 2‐year‐olds (18–24 months, *N* = 72, *M* = 20.71, SD = 2.29, Female = 33). Data from an additional 20 infants were excluded due to: prior experience with tone languages (6), language impairment (1), family history of dyslexia (3), neurodevelopmental disorder (1), preterm birth (1), fussiness/crying during testing (3), age too young or old for the groups (5). The study was approved by the [name suppressed] Human Research Ethics Committee (H14705). Participating families provided informed consent prior to the experiment and received a $30 voucher for their time and travel. The power estimate exceeded that of the prior study on which the current research was based (Kuhl et al. 2003) and was determined in accordance with recommendations for sample size considerations in infant experimental research (Oakes 2017). The current sample size was sufficient to meet the target estimated by an a priori GPower analysis (Faul et al. 2009), which indicated that a medium effect size (*f* ^2^ = 0.15) with *α* = 0.05 and three variables would yield approximately 95% power.

### 2.2 Stimuli and Procedure

**FIGURE 1:**
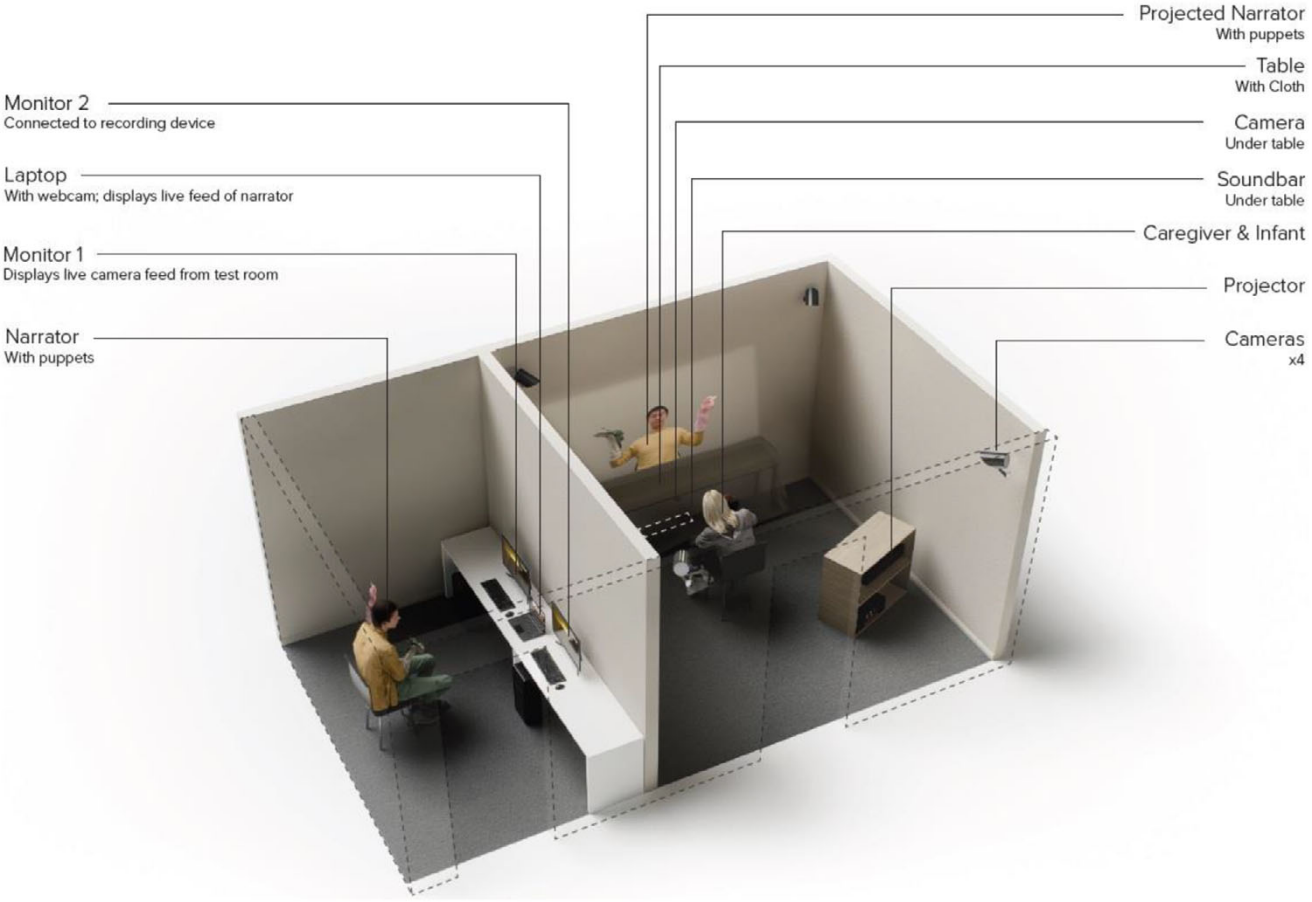
An illustration of the setup for the XR‐Live condition.

The procedure comprised a training phase and two subsequent experimental phases. In each of these phases, infants sat on their caregivers’ laps facing the training or testing stimuli. In training, infants from each age group were randomly assigned to one of the three conditions. Caregivers and infants sat on a chair 1.5 m away from an agent presented to them in real (the Live condition) or virtual (both XR conditions) form. In the Live condition, infants interacted directly with the agent in the same room. In both XR conditions (Figure 1), a BenQ (MX560) projector was linked with a Yamaha (YAS‐109) soundbar hidden underneath a cloth‐covered table. The agent (same size and volume) was projected onto a wall right above the table with audio delivered through a hidden sound bar to mirror natural interaction between the agent and infants across the table. In the XR‐Live condition, a Sony (DCR‐HC28) camera at front, as well as four corner cameras, captured participants’ feedback. The agent interacted with infants from an adjacent room in real‐time. The XR‐Recorded condition adopted the same setting as the XR‐Live condition, but the agents’ speech and actions were pre‐recorded. Therefore, the key distinction between the Live and XR‐Live conditions lay in the medium of delivery: the former involved direct face‐to‐face interaction, whereas the latter involved real‐time interaction through a virtual interface. In contrast, the difference between the XR‐Live and XR‐Recorded conditions concerned contingency: while the XR‐Live condition allowed real‐time responsiveness between the agent and the child, the XR‐Recorded condition presented the same content as a fixed sequence, eliminating contingent responses. To characterize infants’ engagement and contextualize differences in attentional patterns across conditions, we coded the proportion of time infants attended to the agent and puppets during the training phase. This provided a coarse index of infants’ overall attentional engagement with the display. Infants showed higher engagement in the Live condition (Year 1: 83%, Year 2: 81%) compared with the XR‐Live (Year 1: 78%, Year 2: 77%) and XR‐Recorded (Year 1: 70%, Year 2: 64%) conditions.

**FIGURE 2:**
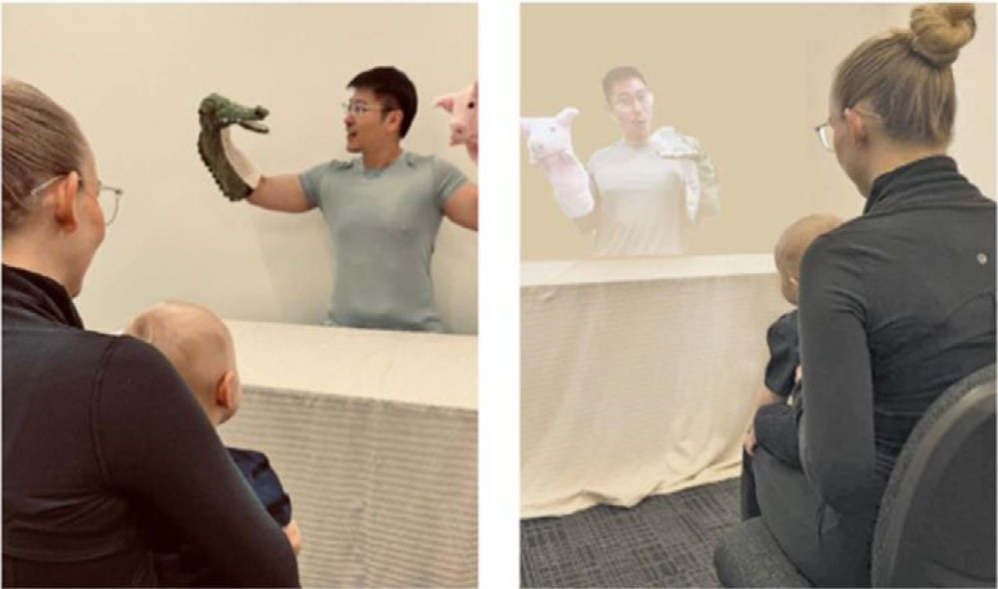
Illustrations of Live (left) and XR‐Live or XR‐Recorded (right) training conditions.

Regardless of conditions, all infants were exposed to the same puppet show involving the same auditory and visual stimuli with matched speech and materials. Visually, infants were shown two distinct hand puppets each paired with one of the two novel words contrasted in Mandarin (rising or dipping) tones as their names (Figure 2). The tone‐bearing syllable used was /boʊ/, resulting in two non‐words phonological licit in Mandarin. The puppet‐tone pairings were counterbalanced across participants. In the puppet show, each pairing was presented 100 times in a set script.

**FIGURE 3:**
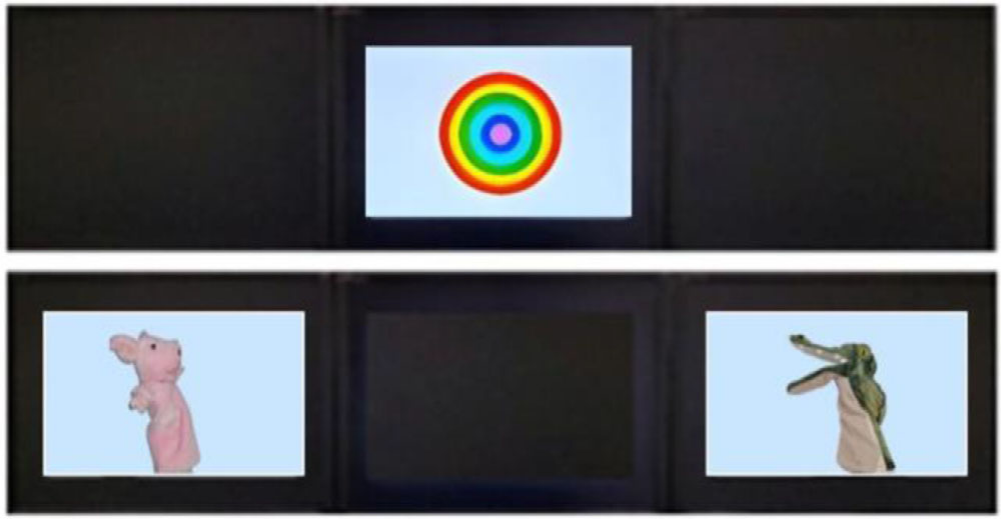
An example of visual stimuli appeared in an object‐label association trial. The bull's eye functions as the attention‐getter. When puppets appeared, the auditory stimulus was /boʊ/ repeated in either the rising or dipping tone in a trial.

To examine the training outcomes, two subsequent experiments were presented. In the object‐label association experiment, infants were presented with four trials (Figure 3). In each trial, a bull's eye was first presented on the central screen as an attention‐getter. After infants focused their attention, both puppets introduced in training were displayed on the left and right monitors. Meanwhile, only one name was displayed (/boʊ/ in rising or dipping tone). Infants’ successful learning was indicated by looking at the puppet that matched the tone they learned in training. Specifically, infants’ looking time (LT) for the “correct” puppet (the one paired with the sound in training) and the “incorrect” puppet (mismatched sound‐puppet association) were recorded. The dependent variable was LT% (toward the correct puppet), computed for each test trial, as follows:

$$\mathrm{LT}\%=\frac{\mathrm{LT}\;\mathrm{correct}\;\mathrm{puppet}\;}{\left(\mathrm{LT}\;\mathrm{correct}\;\mathrm{puppet}+\mathrm{LT}\;\mathrm{incorrect}\;\mathrm{puppet}\right)}$$

Longer LTs to the correct over the incorrect object would result in higher LT% scores and would imply successful learning. The appearances of puppet locations and puppet‐tone associations were counterbalanced across trials and participants. Here, infants’ learning outcomes were separated by learning targets, that is, rising and dipping tones.

**FIGURE 4:**
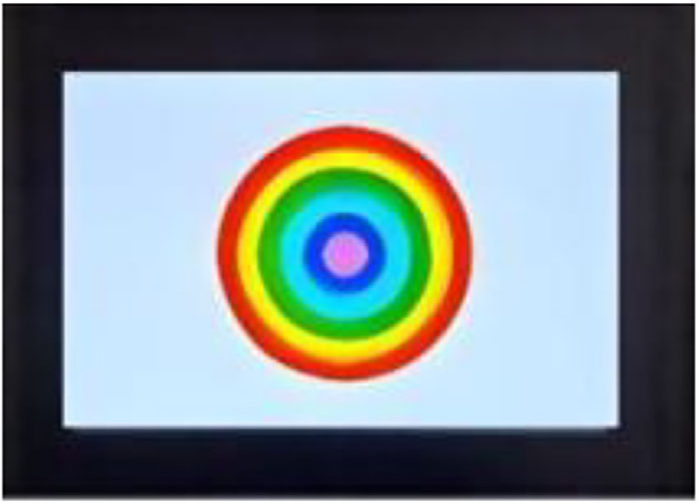
An example of the visual stimulus appeared in a perceptual discrimination trial. The bull's eye functions as the attention‐getter, allowing infants to concentrate on the auditory components of the task.

Following up, a perceptual discrimination experiment was conducted to examine infants’ discrimination ability measuring their perceptibility of non‐native Mandarin tones. In this experiment, infants first underwent a habituation phase where they were repeatedly presented with one tone (e.g., /boʊ/ in rising tone) in each trial. Habituation was considered reached when infants’ average looking time during the last three trials dropped below 50% of the average looking time during the first three trials. Infants showed significant looking time difference (*p* < 0.001) between the first three (*M* = 11,483 ms, SE = 457 ms) and last three (*M* = 5300 ms, SE = 317 ms) trials, indicating overall successful habituation. Once habituated, infants underwent a test phase where they were presented with four trials, including two alternating trials where new tones (different from the habituated tone) were inserted alternating with the habituated tones (e.g., dipping‐rising‐dipping‐rising), and two non‐alternating trials displaying the tone heard in the habituation phase (e.g., rising‐rising‐rising‐rising). When infants looked away for more than 2 s, a trial ended, and the next trial would start. Longer looking times between the two trial types, that is, alternating and non‐alternating trials, indicated successful discrimination. The tones in habituation were counterbalanced across participants. In the perception phase, the visual stimulus was an animated bull's eye consistently appeared on the central screen (Figure 4). Fifteen first‐year and 12 second‐year infants did not complete this experiment due to loss of attention during habituation, and their results were excluded from analyses.

In both experiments, the same equipment was used to present audiovisual stimuli: three 21.5‐inch monitors (Dell P2222H), and two Audioengine (A2+) desktop speakers positioned beside the monitors. A Sony (DCR‐HC28) camcorder recorded infants head‐turn/visual fixation patterns. Caregivers wore headphones and were instructed to keep silent throughout testing to ensure no responses from them to affect infants’ gaze patterns. The experiment was programmed with Habit2 software, which recorded infants' looking behavior as indicators of learning and perception. During testing, the experimenter coded infants' responses in real time, using closed‐circuit video monitoring. To verify the online coding accuracy, experimental outcomes were coded offline by an independent coder. The inter‐rater reliability was high for both perceptual discrimination and (Pearson's *r* = 0.85) and object‐label association (*r* = 0.80) experiments.

## 3 Results

### 3.1 Experiment 1 Object‐Label Association

A lmer model was conducted with Age (2‐level: 6–12 months, 18–24 months), Condition (3‐level: Live, XR‐Live, XR‐Recorded) and Tone (2‐level: rising, dipping) as fixed factors, Participant as the random factor, and LT% at the matched object over total LT as the dependent variable. Tone was an important factor (*F* (1, 534) = 17.92, *p* < 0.001), with the dipping tone (*M* = 57.0%, 95% CI [53.7–60.3]) better learned than the rising tone (*M* = 46.9%, 95% CI [43.6–50.2]). The interaction between Age and Tone (*F* (1, 534) = 7.33, *p* = 0.007) was also significant, and post hoc analyses showed that the dipping tone was better learned than the rising tone for second‐year (estimate = –16.51, SE = 3.39, *df* = 412, *t* = –4.88, *p* < 0.001) but not first‐year (estimate = –3.63, SE = 3.34, *df* = 403, *t* = –1.09, *p* = 0.278) infants (Table 1). Age (*F* (1, 534) = 0.01, *p* = 0.910), Condition (*F* (2, 534) = 0.12, *p* = 0.887), and other interactions (smallest *p* = 0.456) did not reach statistical significance (Table 2).

**TABLE 1:** Mean looking time percentage looking at the correct object when associated with the rising and dipping tones in the test phase across age and condition in the object‐label association experiment.

Age	Condition	Rising	Dipping	Estimate	SE	*df*	*t*	*p*
Year 1	Live	54.7%	53.9%	0.86	5.89	407	0.15	0.884
	XR‐Live	47.9%	55.3%	−7.35	5.70	402	−1.29	0.198
	XR‐Recorded	48.1%	52.5%	−4.40	5.79	400	−0.76	0.448
Year 2	Live	42.0%	60.4%	−18.35	5.83	415	−3.15	0.002
	XR‐Live	45.1%	57.2%	−12.17	6.03	417	−2.02	0.044
	XR‐Recorded	43.6%	62.6%	−19.02	5.73	404	−3.32	0.001

**TABLE 2:** One‐sample *t*‐tests results against (50%) chance level across age and condition and tone in the object‐label association experiment.

Age	Condition	Tone	*t*	*df*	*p*
Year 1	Live	Rising	1.01	43	0.317
		Dipping	0.73	44	0.469
	XR‐Live	Rising	−0.51	46	0.610
		Dipping	1.37	47	0.177
	XR‐Recorded	Rising	−0.63	45	0.531
		Dipping	0.54	45	0.591
Year 2	Live	Rising	−1.78	47	0.082
		Dipping	2.50	42	0.017
	XR‐Live	Rising	−1.18	43	0.245
		Dipping	2.09	40	0.043
	XR‐Recorded	Rising	−1.79	45	0.080
		Dipping	3.62	47	0.001

One‐sample *t*‐tests results of the LT% at the matched object over total LT against (50%) chance level adhere to the main pattern that learning occurred predominantly for the dipping tone among older infants (Table 3), as all conditions showed learning at Year 2, but none at Year 1. Regarding the rising tone, although trends could be seen among 2nd‐year infants, no condition reached significance at either age (Figure 6).

**TABLE 3:** Mean looking times (in msec) for the non‐alternating trials and alternating trials in the test phase across age and condition in the perceptual discrimination experiment.

Age	Condition	Non‐alt trial	Alt trial	Estimate	SE	*df*	*t*	*p*
Year 1	Live	5776	6962	−1186	885	332	−1.34	0.181
	XR‐Live	7165	7983	−818	1080	332	−0.76	0.451
	XR‐Recorded	5712	7610	−1899	1050	336	−1.81	0.071
Year 2	Live	7904	8354	−450	904	332	−0.50	0.619
	XR‐Live	3754	6484	−2729	1060	333	−2.57	0.011
	XR‐Recorded	3675	6176	−2501	1080	333	−2.32	0.021

To understand infants’ levels of interest, another lmer model was run with Age, Condition and Tone as fixed factors, Participant as the random factor, and total LT as the dependent variable. Condition was the only significant factor (*F* (1, 124) = 8.29, *p* < 0.001). Post hoc analyses revealed significant difference between Live (*M* = 6040 ms, SE = 224 ms) and XR‐Live (*M* = 7175 ms, SE = 225 ms) conditions (estimate =  –1135.1, SE = 317, *df* = 137, *t* = –3.58, *p* = 0.001) as well as between Live and XR‐Recorded (*M* = 7134 ms, SE = 222 ms) conditions (estimate = –1094.3, SE = 315, *df* = 137, *t* = –3.47, *p* = 0.002), but not between the two XR conditions (estimate = 40.8, SE = 317, *df* = 136, *t* = 0.13, *p* = 0.991).

### 3.2 Experiment 2 Perceptual Discrimination

**FIGURE 5:**
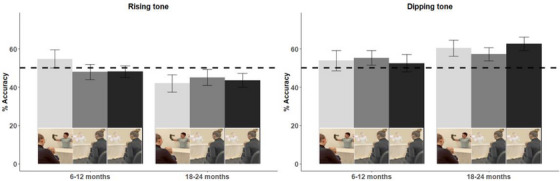
Mean percentage of looking times at the correct puppet when named with different tones in Live (light bar), XR‐Live (grey bar), and XR‐Recorded (dark bar) conditions in Experiment 2 Object‐label Association. Accuracy of 50% indicated chance performance. Error bar = ±1SE.

Data were analyzed using linear mixed‐effects models implemented via the *lme4* package (Bates et al. 2015) in R (version 4.4.1), run through RStudio (version 2025.05.1). Post hoc tests were corrected for multiple comparisons. A lmer model was conducted with Age (2‐level: 6–12 months, 18–24 months), Condition (3‐level: Live, XR‐Live, XR‐Recorded) and TrialType (2‐level, non‐alternating, alternating) as fixed factors, Participant as the random factor, and LT at each trial as the dependent variable. We observed a main effect of TrialType (*F* (1, 332) = 14.91, *p* < 0.001) with longer LT on alternating (*M* = 7261 ms, 95% CI (6449–8073 ms)) than non‐alternating trials (*M* = 5664 ms, 95% CI (4848–6480 ms)), suggesting discrimination between alternating and non‐alternating trials across ages and conditions. The interaction between Age and Condition (*F* (2, 108) = 3.68, *p* = 0.028) was also significant, and post hoc analyses showed differences only among older infants between Live and XR‐Live (estimate = –3010, SE = 1210, *df* = 108, *t* = –2.49, *p* = 0.037) as well as between Live and XR‐Recorded (estimate = –3203, SE = 1210, *df* = 110, *t* = –2.64, *p* < 0.001) conditions, but not among younger infants (largest estimate = 1205, SE = 1220, *df* = 108, *t* = 0.99, *p* = 0.584) infants (Table 1). Age (*F* (1, 108) = 1.29, *p* = 0.259), Condition (*F* (2, 108) = 1.55, *p* = 0.217), and other interactions (smallest *p* = 0.341) did not reach statistical significance. Post hoc analyses showed robust discrimination only at 18–24 months for XR‐Live (estimate = –2729, SE = 1060, *df* = 333, *t* = –2.57, *p* = 0.011) and XR‐Recorded (estimate = –2501, SE = 1080, *df* = 333, *t* = –2.32, *p* = 0.021) conditions (Figure 5).

**FIGURE 6:**
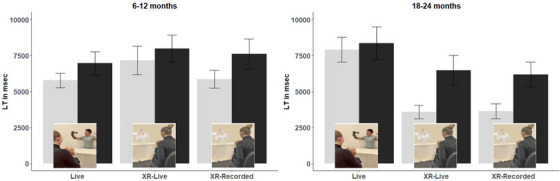
Mean looking times at old (light bar) and new (dark bar) trials across age and condition in Experiment 1 Perceptual Discrimination. Error bar = ±1SE.

## 4 Discussion

The current study pioneered in understanding XR‐infant interaction, exploring factors that affect perception and learning in this process. Outcomes of the perceptual discrimination experiment showed that infants across ages and conditions discriminated new linguistic features after relevant training, although their perception outcomes were modulated by a combination of learners' age and environment. Only older infants showed robust discrimination in both XR conditions, though relatively high LTs were observed in the Live condition. In the object‐label association experiment, infants showed comparable learning outcomes between learning in real‐life and XR environments. Again, their learning ability was modulated by a combination of learners' age and the targets they learn, as older infants showed better learning than younger infants, and better learning of the dipping tone than the rising one.

First and foremost, English‐learning infants exhibited overall sensitivity to a Mandarin tone contrast, laying the perceptual foundation for learning. This finding is in line with prior studies suggesting infants are by no means tone‐deaf but can be sensitive to lexical tones albeit non‐native (Kalashnikova et al. 2024). The rising‐dipping contrast was deemed more difficult than other tone contrasts, evidenced in lowest perceptual sensitivity among listeners across language backgrounds (Huang and Johnson 2011; Liu et al. 2022). Exposure to non‐native tones during training may enhance infants’ perceptual sensitivity. The integration of lexical and associative factors may facilitate discrimination by offering additional cognitive cues that enhance perceptual processing (Yeung et al. 2014). Alternatively, the relative complexity of the employed contrast, while challenging in cognitive effort, may also engage in infants' perceptual systems more intensively (Cheng and Lee 2018).

Moreover, second‐year infants showed more robust discrimination outcomes than first‐year infants. This finding is particularly interesting, as it appears to counter the well‐established decline in sensitivity to non‐native phonetic contrasts after the first year of life due to perceptual attunement (Werker and Hensch 2015). One possible explanation is that certain perceptual abilities are retained or can be reactivated under specific conditions, even beyond the sensitive period. Indeed, studies have shown that infants may show retained or recovered sensitivity to non‐native contrasts (Götz et al. 2018; Liu and Kager 2014). Such sensitivity, along with the lexical learning demands, may result in stronger perceptual outcomes in the second year.

Furthermore, although overall attentional engagement differed somewhat between the two XR conditions, second‐year infants in both XR‐Live and XR‐Recorded conditions showed the strongest discrimination among all groups. On the surface, enhanced discrimination in the XR‐Live condition might appear consistent with accounts highlighting the role of social interaction (e.g., Strouse and Samson 2021). However, the comparable performance in the XR‐Recorded condition indicates that attentional engagement alone does not account for these outcomes. It is possible that other features of the XR environment—such as the life‐sized representation of humans and immersive visual cues—provide sufficient support for perception and learning. Overall, these findings highlight the unique opportunities offered by XR technology for facilitating early learning even in the absence of live interaction.

Our final observation in the perceptual discrimination experiment concerns the performance of second‐year infants in the Live condition, who showed relatively long looking times across both trial types in the test phase. We do not interpret their results as inferior to those in the XR conditions. Rather, infants in the Live condition appeared to sustain uniformly high levels of attention across training and testing phases, consistent with a ceiling effect in which elevated global engagement reduces the variability typically used to index discrimination. In particular, the presence of real face‐to‐face interaction may heighten overall attentional levels (Csibra and Gergely 2006), thereby masking trial‐type differences that would otherwise signal discrimination.

Despite efforts to equate the Live and XR‐Live conditions, subtle behavioral differences may have remained—for instance, more eye contact or contingent cues in the Live condition—which could partly account for the observed differences in older infants’ tone discrimination. Another possible factor is a *novelty effect* associated with the XR environments, where unfamiliarity may have temporarily heightened attention and engagement, enhancing learning. If so, the difference between Live and XR conditions may lessen with increased exposure. Future research should examine how familiarity with XR and agent behavior jointly shape infants’ learning outcomes.

In the object‐label association experiment, second‐year infants demonstrated superior learning compared to first‐year infants, with greater success observed for the dipping tone relative to the rising tone. The observed pattern aligns with the findings from the perceptual discrimination task, where older infants also exhibited stronger discrimination abilities. On the surface, the enhanced performance in the second year appears to reflect normative developmental trajectories: as infants consolidate their native phonological systems in the first year, they gain more robust lexical representations and experience a rapid increase in vocabulary in the second year (Swingley 2009; Werker and Yeung 2005). However, in contrast to prior studies reporting a decline in infants’ associative learning ability of non‐native tones across ages (Hay et al. 2015; Liu and Kager 2018; Singh et al. 2014), the current findings suggest an upward trend. This pattern once again diverges from predictions made by critical period or perceptual attunement frameworks (e.g., Werker and Hensch 2015). It could be that second‐year infants possess more advanced cognitive abilities, enabling them to comprehend object‐label mappings across diverse presentation formats, including XR. This may also speak to the broader literature on screen‐based learning, where young infants’ challenges are often attributed to limited symbolic understanding and transfer deficits (e.g., DeLoache et al. 2010; Troseth et al. 2006).

The substantial difference in learning outcomes across target tones merits further discussion. Infants showed stronger learning performance when the target was a dipping tone but not a rising one. This finding contrasts with prior assumptions, which typically classify dipping tones as more difficult to acquire and rising tones as more accessible—even for non‐native listeners (Hay et al. 2019). We propose that the dipping tone's complex pitch contour may enhance its acoustic salience, thereby facilitating learning. In contrast, the rising tone may pose a greater cognitive challenge for English‐learning infants, as its pitch trajectory overlaps with that of interrogative intonation in English. This acoustic overlap may lead infants to interpret the rising tone at a phrasal rather than lexical level, complicating its integration as a meaningful word cue. This interpretation aligns with perceptual frameworks (e.g., Perceptual Assimilation Model, Best 1994) and suggests a possible interference or transfer effect from the native language (e.g., So and Best 2011). Overall, these results indicate that the tonal properties of the learning target significantly influence associative word learning outcomes.

An interesting finding emerged when considering infants’ total LT, which arguably reflects their level of interest. Infants exposed to the two XR conditions appeared more engaged in the task than those in the Live condition. This may indicate a familiarity effect towards technology carried over from training to the main experiment. In conjunction with the perception task findings—where a potential novelty effect of the XR environments was observed—these results suggest that the medium of presentation may play a dynamic role in shaping early learning experiences, alongside factors related to agents, learning targets, and learners.

Finally, comparable learning outcomes were observed across the Live, XR‐Live, and XR‐Recorded conditions. This promising result highlights the potential for effective information processing and retrieval within XR environments, extending XR‐mediated learning (Si 2015) to a younger population in childhood, supporting the integration of XR as a feasible medium in infancy. Furthermore, XR technologies provide a unique opportunity to overcome barriers of geographic and socio‐economic inequality by offering scalable and widely accessible educational interventions. Early implementation of such technologies could particularly benefit underserved populations, fostering equitable cognitive and linguistic development opportunities, thus directly supporting broader sustainable developmental goals. Having said that, caution is warranted regarding the appropriateness of the learning targets and the developmental stage of the learners.

## 5 Conclusion

This study offers a novel examination of infants’ perception and learning in XR environments, along with key modulating factors. The findings demonstrate that infants are capable of perceiving and acquiring new information in XR contexts, with age and the nature of the learning targets influencing outcomes. Although findings provide limited evidence supporting the unique contribution of specific XR features such as vividness and interactivity (Rauschnabel et al. 2022; Steuer 1992), results suggest a more general facilitative effect of XR environments on early learning. Notably, this effect appears to be robust against traditionally emphasized factors and theoretical frameworks, such as the need for a physically present agent or the constraints proposed by the critical period hypothesis.

Taken together, although there remains room for refinement, increasingly immersive XR technologies show considerable promise as tools for early childhood learning. Adopting XR technologies early in developmental contexts holds promise not only for immediate perceptual and linguistic outcomes but also for long‐term sustainability in education and cognitive development. As outlined in the United Nations’ sustainability agenda, leveraging innovative technologies like XR could significantly reduce developmental inequalities and improve educational access, offering consistent, high‐quality learning experiences regardless of geographic or socio‐economic constraints. Policy‐level integration of XR technologies into educational frameworks should be actively pursued, with recommended guidelines addressing ethical standards, accessibility, environmental sustainability, and pedagogical effectiveness. Collaborative efforts involving researchers, educators, policymakers, and technologists are crucial to realizing XR's full potential as a sustainable developmental tool.

Future research should build upon these findings by directly comparing XR‐mediated learning with that facilitated through traditional screen media, as well as across various XR formats (e.g., virtual vs. augmented reality), agent types (e.g., 2D vs. holographic), and modes of delivery (e.g., human vs. AI‐prompted speech). Additionally, comparative studies involving typically developing infants and those with developmental disabilities will be essential for a more comprehensive understanding of XR's potential in health and in education. Future research should also explicitly focus on longitudinal outcomes of XR‐mediated learning environments, assessing not only immediate educational efficacy but also longer‐term neural, cognitive, linguistic, social, and environmental impacts. Such research aligns directly with and supports global sustainable development goals, providing essential evidence for policymakers and educators.

## Conflicts of Interest

The authors declare no conflicts of interest.

## Data Availability

Anonymous data will be shared upon request.
